# Physical and psychological health at adolescence and home care use later in life

**DOI:** 10.1371/journal.pone.0261078

**Published:** 2021-12-08

**Authors:** Govert E. Bijwaard, Rob Alessie, Viola Angelini, L. H. Lumey

**Affiliations:** 1 Netherlands Interdisciplinary Demographic Institute, NIDI-KNAW/University of Groningen, Groningen, The Netherlands; 2 Department of Economics, Econometrics and Finance, University of Groningen, Groningen, The Netherlands; 3 Department of Epidemiology, Mailman School of Public Health, Columbia University, New York, NY, United States of America; University of South Florida, UNITED STATES

## Abstract

**Objectives:**

To examine the relation between physical and psychological health indicators at adolescence (age 18) and household, personal, and nursing home care use later in life at ages 57–69 years.

**Methods:**

Using medical examinations on men born in 1944–1947 who were evaluated for military service at age 18 in the Netherlands, we link physical and psychological health assessments to national administrative microdata on the use of home care services at ages 57–69 years. We postulate a panel probit model for home care use over these years. In the analyses, we account for selective survival through correlated panel probit models.

**Results:**

Poor mental health and being overweight at age 18 are important predictors of later life home care use. Home care use at ages 57–69 years is also highly related to and interacts with father’s socioeconomic status and recruits’ education at age 18.

**Discussion:**

Specific health characteristics identified at age 18 are highly related to the later utilization of home-care at age 57–69 years. Some characteristics may be amenable to early life health interventions to decrease the future costs of long-term home care.

## 1. Introduction

All developed societies have been going through major demographic changes in the last decades. Improvements in medical science have led to unprecedented increases in life expectancy. These changes and improvements in conjunction with low birth rates have resulted in a progressively ageing society. The demographic changes increase health care costs and threaten the sustainability of long-term care systems. From both an academic and a policy perspective, it is therefore crucial to understand the determinants of long-term care use.

A large literature in both economics and epidemiology suggests that conditions experienced early in life may have a long-term effect on health [1–5]. In particular, adverse early life conditions can have a negative impact on mortality [6–9] cardiovascular disease [10] and cognition [11]. Many illnesses are inherently chronic and long-lasting, leading to persistent health problems and the need for home care. However, some health problems may be persistent when poor health in youth sets in motion trajectories of health-related disadvantages and health and socio-economic risks that may accumulate over the life course [12].

One of the main indicators of later-life poor health is the need for long term care. Despite the potential long-lasting impact of poor health in life, we are not aware of studies that compare health problems in adolescence with home care use at older ages.

In an earlier study in The Netherlands, we found increased long-term mortality in men who at age 18 were more overweight (BMI ≥ 25). These men also had lower SES, as indicated by their education level and their fathers had a lower occupation level [6]. We reasoned that the increased mortality in this group could be a reflection of impaired physical health and a greater need for supportive services in later age, prior to increased mortality. This was our main hypothesis. In view of prior findings, we would need to examine if the association was moderated by SES and education, (or IQ) and also control for age. For mental health we had no prior observations for testing as this characteristic had not been examined before in this group in relation to mortality.

The main contribution of this paper is that we compare findings on standardized military service examinations of men aged 18 to study the association between selected physical health characteristics, in multiple domains, at age 18 and an indicator of mental health, and the utilization of home-based formal care services later in life (around age 60–69), using national Dutch administrative microdata. In the Netherlands, the government encourages people to stay at home for as long as possible by providing formal care in the home setting. A large share of long-term care is provided through formal home care, which has been increasing in recent years [13]. The Netherlands has one of the most extensive public long-term care systems in the world. It has a separate mandatory public insurance system for legal entitlements to formal home care use which covers 100% of the population. This insurance covers all chronic care and included a broad range of home care services for older individuals. Users have to pay a copayment, which depends on the type of care and the amount of care used. The monthly copayment is maximized, depending on income and financial wealth, guaranteeing that long-term care was accessible for all income groups.

We use administrative microdata on Dutch men who were born in 1944–1947 and were examined for military service between 1961–1965. The records include a standardized recording of demographic and socio-economic characteristics including education, father’s occupation, along with a standardized psychometric test battery. At the military examination, all conscripts were scored on seven health aspects of relevance for military service, comprising general health, hearing, eyesight, upper- and lower extremities, intelligence and mental stability. Conscripts deemed unfit on any of these elements were deemed to be unfit for military service. We link these data to national data from the death register and data on persons who received home-based formal care services in the period 2004–2013 to investigate the relationship between measurements of poor health at age 18 and the use of these services.

This paper contributes to the literature in three main ways. First, our measures of early life conditions include health conditions at the beginning of adult life (around age 18). Second, to the best of our knowledge, we are the first to analyse the relationship between early-life health and home care use later in life. Third, thanks to the availability of data on mortality and the use of home-based formal care services over 10 years, we can account for selective survival in our econometric model. This makes it possible to predict home care use based on health measurements at adolescence.

## 2. Data

We combine several sources of data to arrive at the analytical sample. We start from the universe of male recruits from the nationwide Dutch Military Service Conscription Register for the years 1961–1965 and born in the years 1944–1947 (n≈400,000). At the time all men, except those living in psychiatric institutions or in nursing institutes for the blind or for the deaf-mute, were called to the military service induction exam at age 18. These data were previously used for the investigation of the relationship between prenatal famine exposure and obesity in adolescence [6, 14, 15] used a sample to study the relationship between prenatal famine exposure and mortality. This sample included: all the 25,283 men born in the Western Netherlands between November 1944 and March 1946 (famine exposed individuals), a random 15% sample (n = 10,667) of men born before November 1944 or after March 1946 in these same cities, and a random 3% (n = 9,087) of men born between 1944 and 1947 in the remainder of the country. In the analyses we account for this oversampling of famine exposed men through weighting. The final sample extracted from the military examination data for further linkage (via unique personal identification number) included 45,037 men.

**Fig 1:**
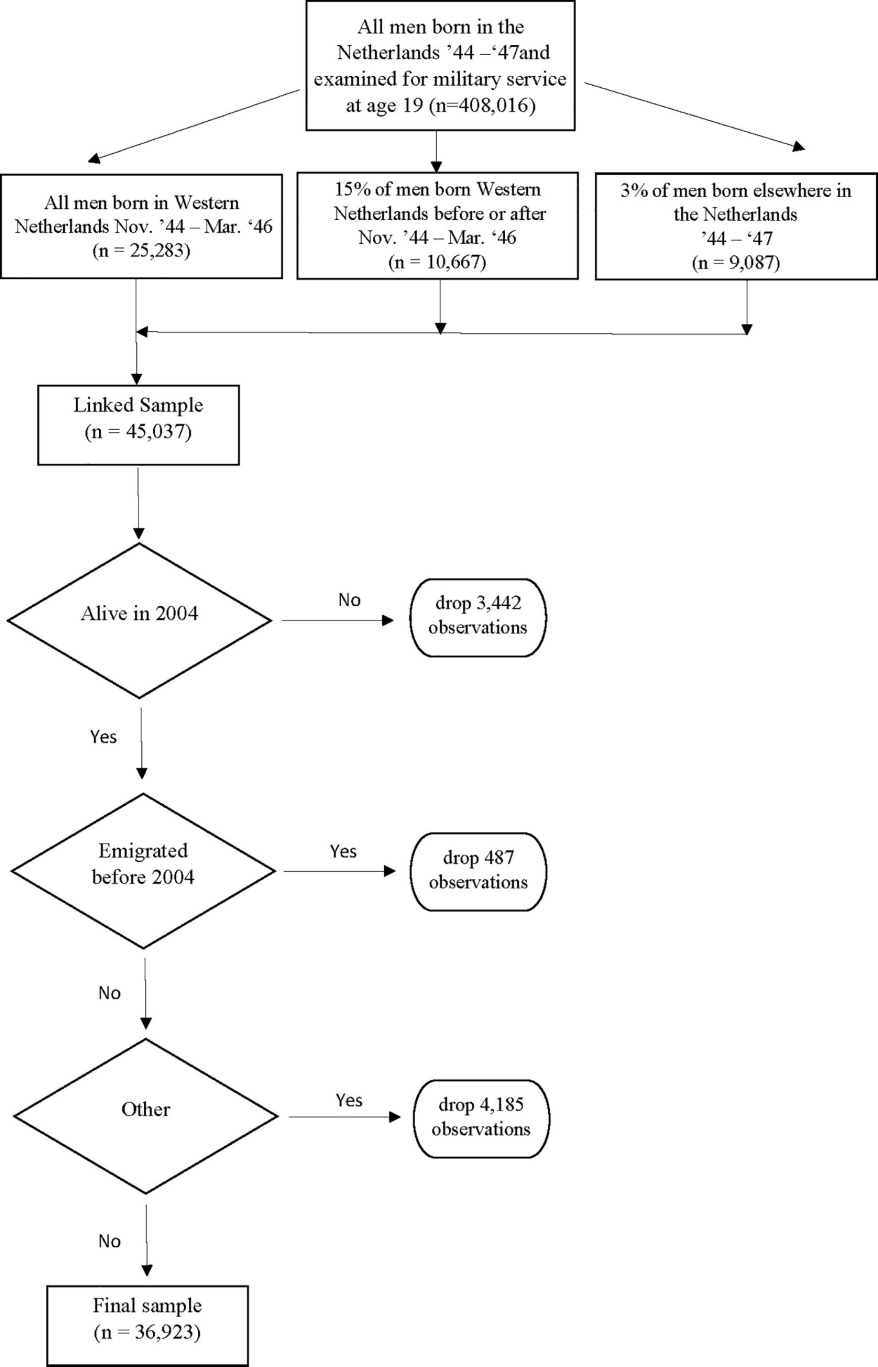
Flowchart.

These data were linked to administrative data available through the secured remote data access system of Statistics Netherlands. We were able to link 36,923 men with home care utilization status in 2004, the first year this information was recorded. 8,114 were lost from the sample between the start of the administrative registers in 1999 and 2004 to follow-up. Of the missing individuals 3,442 are known to have died, 487 to have emigrated and 4,185 were lost due to other reasons, see Fig 1.

Conscripts were scored (1 = fit; 2 = fairly fit; 5 = unfit) on seven health characteristics of relevance for military service, and were combined into an ABOHZIS score. (A: Algemeen = General health; B: Bovenste extremiteit = Upper extremities; O: Onderste extremiteit = Lower extremities; H: Horen = Hearing; Z: Zien = Eyesight; I: Intelligentie = Intelligence; and S: Stabiliteit = Mental Stability). For each measurement we define as poor health any measurement that was less than fit. These measurements were available for 98% of the conscripts. Conscripts deemed unfit on any of these characteristics were declared to be unfit for military service (the unfit-for-service indicator) The most common characteristics with poor score, were: Poor Eyesight (15.0%), poor general health (10.0%), poor Lower extremities (8.0%), and poor Mental health (4.5%). Poor health indication on other scores were less common: Poor Hearing (2.2%) and poor Upper extremities (0.9%) was less common.

In the literature excess weight has also been identified as predictor of later health. [1, 2, 16]. We therefore also included an overweight measure (BMI > 25) from the military examination as a possible predictor of late life home care. At the military examination, a standardized recording of demographic and socioeconomic characteristics including education and father’s occupation was also completed. The military examination included a standardized Raven Progressive Matrices psychometric test as an indicator of intelligence.

Requests for formal home care are evaluated by the Centre of indication-setting Health Care (CIZ). The CIZ has divided the Netherlands into 26 regional care purchasing agencies (`Zorgregiokantoor’) that assess the eligibility for home care use. The decision is based on functional limitations of the applicant and health status, but not on income or wealth. In the analysis, potential regional differences in the access and financing of home care are taken into account by linking individuals to their regional health care agency and a dummy variable adjustment for region. Statistics Netherlands distinguishes four categories of home care (non-residential care for which the expenditures are covered by the public insurance system): 1) *household care*: if an individual has received household assistance, such as cleaning and food preparation, which is partly paid through the `Wet Maatschappelijke Ondersteuning (WMO)’, the social support act; 2*) personal care*: if an individual has received personal care for which the expenditures are covered by `Algemene Wet Bijzondere Ziektekosten (AWBZ)’, General law on special sickness costs, such as aid with dressing, washing, eating and, drinking; 3) *nursing care*: if an individual has received nursing care for which the expenditures are covered by `Algemene Wet Bijzondere Ziektekosten (AWBZ)’, such as nursing, aid in medication use or injections and 4) *total care*: if an individual has received any of the three home care categories. For each individual, the use of any home care in a particular year was recorded. At the start of 2004, the first year of home care use observation, we observed that 0.8% of the men received household care, 0.9% personal care, 1.0% nursing care, and 1.7% any of these three categories of home care use (total care). The use increased to 1.6% for household care, 1.9% for personal care, 1.4% for nursing care, and 3.4% for total care in 2013, the final year of observation of home care use. We will analyse the three different home care categories and the total home care use separately. Between 2004 and 2013, 3437 men died and 586 were lost due to other reasons. Table 1 reports the average value of the health indicators and background variables and its relation to home care use.

**Table 1:** Descriptive statistics–conditions at age 18.

	All	Home care use proportion in 2004			
		Household	Personal	Nursing	Total
Unfit for service	0.127	0.272	0.229	0.166	0.209
Overweight (BMI >25)	0.063	0.098	0.115	0.068	0.080
Poor general health	0.132	0.315	0.235	0.161	0.208
Poor sight	0.171	0.262	0.232	0.151	0.213
Poor mental health	0.067	0.220	0.210	0.110	0.175
Poor upper extremity	0.031	0.147	0.109	0.068	0.103
Poor lower extremity	0.106	0.285	0.269	0.198	0.215
Poor hearing	0.044	0.180	0.127	0.082	0.117
*father’s occupation*					
professional^a^	0.144	0.140	0.116	0.098	0.117
White collar	0.264	0.221	0.165	0.190	0.213
Farm owner	0.102	0.078	0.153	0.157	0.131
skilled	0.267	0.250	0.264	0.268	0.246
unskilled	0.160	0.183	0.207	0.165	0.171
unknown	0.064	0.129	0.096	0.126	0.123
*IQ*					
1 (highest)	0.179	0.076	0.065	0.098	0.091
2	0.283	0.165	0.186	0.237	0.226
3^a^	0.204	0.218	0.234	0.222	0.200
4	0.141	0.113	0.142	0.118	0.115
5	0.093	0.162	0.140	0.171	0.161
6 (lowest)	0.045	0.107	0.107	0.071	0.097
9 (missing)	0.048	0.159	0.127	0.082	0.112
*Education level*					
Low	0.211	0.358	0.359	0.331	0.345
Medium	0.664	0.541	0.567	0.583	0.545
High	0.124	0.101	0.075	0.086	0.111
N =	36,923	305	332	368	628

Weighted by sampling weight. All: the whole sample; Household: men using household home care in 2004; Personal: men using personal home care in 2004; Nursing: men using nursing home care in 2004; Total: men using any home care in 2004.

^a^ Baseline category in all analyses: father’s occupation professional and IQ-level 3.

**Fig 2:**
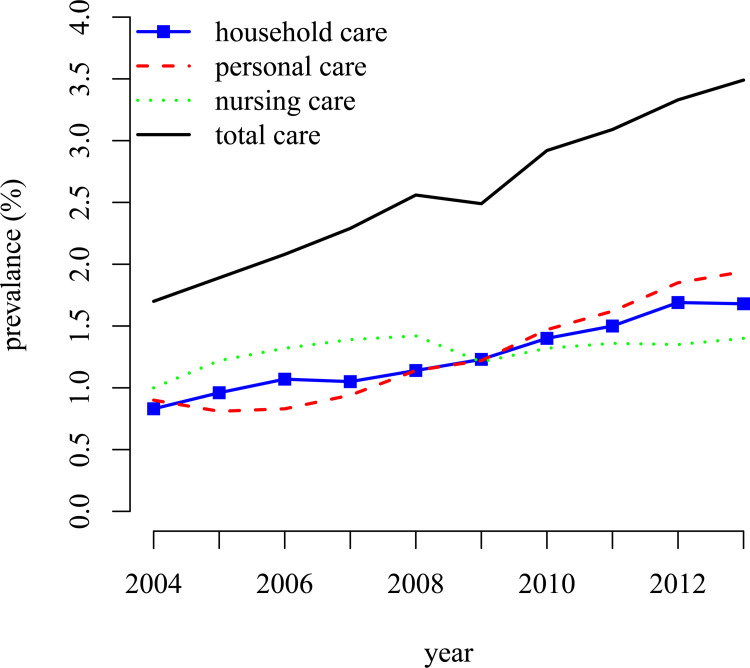
Development of home care use 2004–2013.

**Fig 3:**
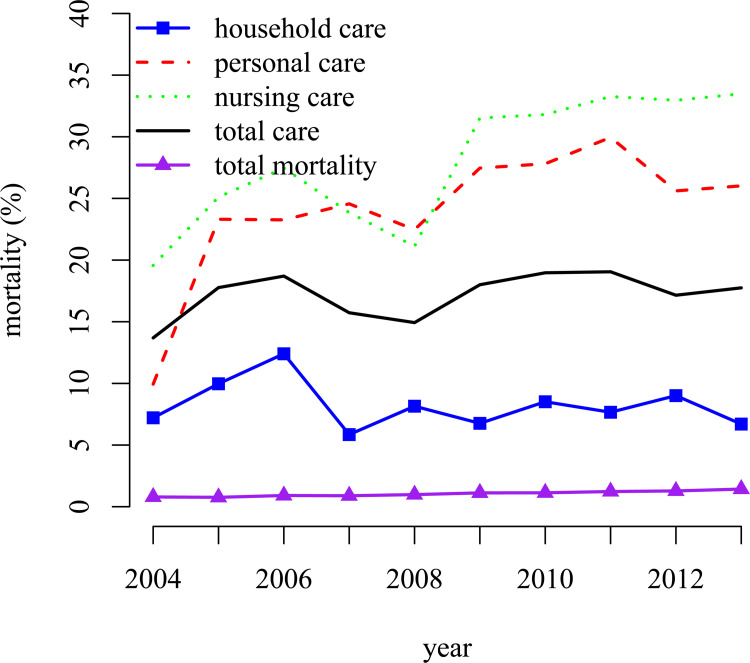
Mortality by home care use in each year.

Fig 2 clearly shows that the use of home care is increasing over the years (i.e it is increasing with age). The increase is most pronounced for personal care. Fig 3 shows that there is a strong positive correlation between mortality and home care use and that for personal care and nursing care the mortality is increasing with age. This emphasizes the importance to account for selective mortality (see also the S1 Table).

Based on the home address of each individual, available for every year, we know which care purchasing agency was responsible for the decision to allow provide home care to specific individuals.

## 3. Methodology

The anonymity of the included individuals is guaranteed by Statistics Netherlands. The data can only be analyzed at Statistics Netherlands or through Remote Access. Access to the data is only possible with fingerprint ID and the personal smartcard. The study was reviewed by the Institutional Review Board of the Columbia University Medical Center in New York, NY. The Board determined that studies on this study population do not meet the DHSS definition of ’human subjects research’ and are exempt from IRB approval. In the Netherlands, the study does not need approval by Ethical Review Boards or by the National Data Protection Authority (College Bescherming Persoonsgegevens) as all study procedures are in compliance with Dutch privacy legislation and do not need the consent of the data subjects concerned or of their relatives. The study is based on population wide administrative records and not on patient records.

We seek to find the association between early-life health measurements and home care use later in life. We postulate the following panel probit model for home care use.

$$h_{it}^{*}=\beta_{x}^{\prime }x_{i}+\beta_{t}+\eta_{i}+\epsilon_{it}\;i=1,\ldots ,N;\;t=2004,\ldots ,2013$$ (1)

where $h_{it}^{*}$ represents a latent variable of the observed home care indicator; *x*_*i*_ is a vector of time-invariant regressors (measured at age 18), β_*t*_ is a period effect, η_*i*_ is an unobserved individual effect capturing unobserved heterogeneity and ϵ_*it*_ denote standard normally distributed, serially uncorrelated error term assumed to be independent of *x*_*i*_. An individual is observed using home care in year *t = 2004*,*…*,*2013*, *h*_*it*_ = 1, when $h_{it}^{*}>0$.

An issue is that selective survival/attrition which may depend on home care use in the previous period (see Fig 2) may distort the analyses. We therefore also include a probit model for survival

$$d_{it}^{*}=\alpha_{h}h_{i,t-1}+\alpha_{x}^{\prime }x_{i}+\alpha_{t}+\theta_{i}{+v}_{it}\;i=1,\ldots ,N;\;t=2003,\ldots ,2013$$ (2)

where $d_{it}^{*}$ represents a latent variable of the observed mortality indicator; *α*_*h*_ captures the effect of home care use in the preceding period on mortality; *α*_*x*_ captures the effect of time-invariant regressors on mortality, α_*t*_ is a period effect and, θ_*i*_ is an unobserved individual effect, and *v*_*it*_ denote standard normally distributed, serially uncorrelated error term assumed to be independent of *x*_*i*_. For the initial period (*t = 2003*, before home care use is observed,) and the first period, *t = 2004*, we assume a model including only the time-invariant regressors and a period-specific intercept, α_*t*_. An individual has died in year *t* when $d_{it}^{*}>0$. We have selective survival by allowing the individual heterogeneity terms of the survival equation, θ_*i*_, to be correlated with the individual heterogeneity terms of the home care use equation, η_*i*_, with ρ = Corr(θ_*i*_, η_*i*_), i.e assuming a bivariate normal distribution for unobserved heterogeneity. The joint likelihood is estimated using the STATA procedure cmp [17].

For each of the four home care use categories, we estimate a panel probit model and the bivariate panel probit model accounting for selective survival. We also estimate models that includes an interaction between the health measurements and the binary (manual: self-employed, unskilled and skilled vs non-manual: professional and clerical) indicator of the father’s occupation or with the discrete education level indicator (low, middle, high).

In all analyses we account for oversampling of the famine exposed men using weighted estimation with weights equal to the probability to be sampled, specifically the weight was one for the famine exposed men, 1/0.15 for men born before November 1944 or after March 1946 in the famine region, and 1/0.03 for men born in the remainder of the country.

## 4. Results

First, we investigate whether the unfit-for-service indicator at the military examination predicts home care use later in life, taking into account other characteristics that may influence home care use: father’s occupation (in six categories), IQ measurement at age 18, a dummy for the care purchasing agency region and, a quadratic trend in the month of birth. Father’s occupation measures the socio-economic position of the household at age 18. Intelligence, as measured by an IQ-test, affects both health [18–20] and the take-up rate of home care use. We do not include the education level, because education is likely to be an endogenous variable. The quadratic trend in the month of birth is included to capture the age effect on home care use (together with the period effect). The estimated odds ratios are reported in Table 2. The full estimation results are available upon request. This unfit-for-service indicator clearly predicts home care use later in life. Accounting for selective survival hardly influence the estimation results.

**Table 2:** Odds ratios panel probit model of unfit for service at military examination on later life home care use.

Household	Personal	Nursing	Total
2.230**	1.138^+^	1.129**	1.259**
(0.216)	(0.062)	(0.047)	(0.059)
*Accounting for selective survival*			
2.186**	1.118**	1.109**	1.229**
(0.207)	(0.047)	(0.036)	(0.051)
Average prevalence			
1.06%	1.20%	1.21%	2.31%

Also included are a quadratic trend in the birth date, period dummies for the home care observation year and care purchasing agency region dummies, father’s occupation and IQ-measurement. All analyses weighted by the sampling weights.

^+^*p <* 0.05

** *p <* 0.01. Household: men using household home care in 2004; Personal: men using personal home care in 2004; Nursing: men using nursing home care in 2004; Total: men using any home care in 2004.

Next, we estimate models which include the seven health indicators measured at age 18,: overweight (BMI > = 25), poor general health, poor eyesight, poor mental health, poor upper extremity, poor lower extremity, and poor hearing. Again, we also include in our models father’s occupation, IQ measurement at age 18, care purchasing agency region, and a quadratic trend in the month of birth.

The reported estimated odds ratios of health measurements on homecare use in Table 3 suggest that healthy ageing starts early in life. The estimated odd’s ratios of other control variables can be found in the S2 Table and the full table with coefficients in the S3 Table and the S4 Table. Poor mental health at age 18 increases the probability of all types of home care use: males scoring a ’fair’ or ’unfit’ have a 1.5 times higher probability to receive total care 50 years later compared to men scoring `fit’ on mental health and 3 times higher probability to receive household care. Being overweight at age 18 increased the risk of later life home care use by 30%. General health problems early in life and problems with legs or feet increase the probability of home care use only somewhat (1.1 times). Other smaller effects are seen for Poor eyesight at age 18 which increases the probability of the need for household and personal care and for poor hearing at age 18 which increases the need for nursing care. Comparing the results in the upper panel of Table 3 with the results in the lower panel shows that accounting for selective survival lowers the estimated impact of the health measurements at age 18 on home care use later in life.

**Table 3:** Odds ratios panel probit model of early life health on later life home care use.

	Household	Personal	Nursing	Total
Overweight	1.173	1.306**	1.333**	1.394**
	(0.131)	(0.087)	(0.066)	(0.080)
Poor general health	1.640**	1.042	1.055	1.132^+^
	(0.162)	(0.060)	(0.046)	(0.057)
Poor eyesight	1.243+	1.140**	1.030	1.062
	(0.113)	(0.054)	(0.029)	(0.046)
Poor mental health	2.962**	1.600**	1.326**	1.566**
	(0.330)	(0.112)	(0.075)	(0.099)
Poor upper extremity	0.713	0.767	0.873	0.904
	(0.216)	(0.112)	(0.096)	(0.115)
Poor lower extremity	1.818**	1.148^+^	1.083	1.137^+^
	(0.227)	(0.074)	(0.053)	(0.065)
Poor hearing	0.956	1.111	1.266**	1.219
	(0.235)	(0.121)	(0.101)	(0.115)
*Accounting for selective survival*				
Overweight	1.216	1.239**	1.271**	1.324**
	(0.135)	(0.063)	(0.050)	(0.067)
Poor general health	1.609**	1.037	1.044	1.120**
	(0.157)	(0.045)	(0.036)	(0.049)
Poor eyesight	1.219+	1.106**	1.025	1.055
	(0.106)	(0.040)	(0.029)	(0.039)
Poor mental health	2.881**	1.470**	1.273**	1.518**
	(0.314)	(0.081)	(0.057)	(0.085)
Poor upper extremity	1.177	0.830	0.919	0.916
	(0.269)	(0.092)	(0.080)	(0.100)
Poor lower extremity	1.611**	1.128^+^	1.083^+^	1.129^+^
	(0.200)	(0.055)	(0.042)	(0.056)
Poor hearing	0.686	1.081	1.192**	1.175+
	(0.153)	(0.091)	(0.076)	(0.097)

Also included are a quadratic trend in the birth date, period dummies for the home care observation year and care purchasing agency region dummies, father’s occupation and IQ-measurement. All analyses weighted by the sampling weights.

^+^*p*<0.05,**p<0.01 Household: men using household home care in 2004; Personal: men using personal home care in 2004; Nursing: men using nursing home care in 2004; Total: men using any home care in 2004.

Socioeconomic status early in life has a long-lasting effect on health [2, 4, 21–23] leading to changes in home care use later in life. This is reflected in the statistical significance of the marginal effects of father’s occupation on home care use (S1 Table). However, the socioeconomic status is also directly related to health early in life, the health measurements at age 18 we use. We therefore also estimate a model that includes an interaction between the health measurements and the binary (manual: self-employed, unskilled and skilled vs non-manual: professional and clerical) indicator of the father’s occupation The resulting odd’s ratios of the health measurements on home care later in life, shown in Table 4 indicate that the impact of early health measurements on home care use differs substantially by socioeconomic status. Whereas the impact of poor general health on home care use is larger for men from lower socioeconomic background (father with manual occupation), the impact of overweight and poor mental health is larger for men from higher socioeconomic background (father with non-manual occupation).

**Table 4:** Odds ratios panel probit model of early life health on later life home care use accounting for selective survival, manual vs non-manual father’s occupation (interaction).

	Household	Personal	Nursing	Total
	*Father’s occupation manual*			
Average prevalence	1.19%	1.29%	1.28%	2.53%
Overweight	1.400^+^	1.313**	1.275**	1.302**
	(0.238)	(0.091)	(0.069)	(0.092)
Poor general health	2.095**	1.023	0.998	1.150+
	(0.264)	(0.064)	(0.050)	(0.071)
Poor eyesight	1.206	1.089	1.068	1.083
	(0.192)	(0.059)	(0.045)	(0.059)
Poor mental health	2.547**	1.398**	1.282**	1.418**
	(0.341)	(0.109)	(0.081)	(0.113)
Poor upper extremity	1.158	0.737	0.953	0.913
	(0.419)	(0.137)	(0.132)	(0.159)
Poor lower extremity	1.976**	1.254**	1.163+	1.203**
	(0.262)	(0.086)	(0.062)	(0.085)
Poor hearing	0.494**	1.070	1.184	1.145
	(0.113)	(0.128)	(0.106)	(0.133)
	*Father’s occupation non-manual*			
Average prevalence	0.81%	0.86%	0.92%	1.76%
Overweight	1.556	1.202+	1.273**	1.388**
	(0.401)	(0.101)	(0.081)	(0.114)
Poor general health	1.425	1.101	1.103	1.143^+^
	(0.229)	(0.074)	(0.058)	(0.077)
Poor eyesight	1.288^+^	1.111^+^	0.992	1.019
	(0.133)	(0.059)	(0.042)	(0.056)
Poor mental health	5.020**	1.616**	1.305**	1.696**
	(0.813)	(0.143)	(0.096)	(0.153)
Poor upper extremity	1.690^+^	1.005	0.825	0.970
	(0.432)	(0.197)	(0.141)	(0.196)
Poor lower extremity	1.014	0.961	1.002	1.058
	(0.139)	(0.078)	(0.062)	(0.084)
Poor hearing	1.361	1.028	1.120	1.183
	(0.452)	(0.155)	(0.129)	(0.174)

Also included are a quadratic trend in the birth date, period dummies for the home care observation year and care purchasing agency region dummies, father’s occupation and IQ-measurement.

^+^*p*<0.05,**p<0.01 Household: men using household home care in 2004; Personal: men using personal home care in 2004; Nursing: men using nursing home care in 2004; Total: men using any home care in 2004.

It is well known that the education level of an individual is an important factor in predicting home care use later in life [24]. However, education is also likely to be endogenous, as poor health limits educational progress and because confounding factors may influence both education choice and health later in life [25–27]. For this reason, we did not include education in the control variables and did not estimate a model with interaction between the education level and the health measurements, similar to the interaction model for father’s occupation. A separate analysis by education level suffers less from this endogeneity.

Educational attainment is observed at the military examination at age 18 for each individual and we group the individuals into three levels: low, medium and high education, depending on the type of high school they attended. At the time the individuals in the study went to school the education system in the Netherlands was characterised by a minimum school-leaving age of 14.

The resulting odds ratios of the health measurements on home care use later in life, reported in Table 5, indicate substantial differences by education level. The impact of poor mental health in adolescence on home care use later in life seems much larger for the high educated. Note that poor mental health is very rare amongst the high educated men. Being overweight and poor general health have the largest impact on home care use for the less educated men. Problems with upper extremities, arms or hands, and lower extremities, legs or feet, only affect the home care probability of the less educated.

**Table 5:** Odds ratios panel probit model of early life health on later life home care use accounting for selective survival, by education level (separate models).

	Household	Personal	Nursing	Total
	*Low education level*			
Average prevalence	1.99%	2.18%	2.09%	1.99%
Overweight	3.765**	1.456**	1.296**	1.452**
	(0.565)	(0.164)	(0.111)	(0.157)
Poor general health	2.969**	1.051	1.081	1.170
	(0.396)	(0.151)	(0.080)	(0.172)
Poor eyesight	1.052	1.221^+^	1.047	1.139
	(0.152)	(0.122)	(0.080)	(0.187)
Poor mental health	1.159	1.451**	1.252**	1.493**
	(0.139)	(0.152)	(0.104)	(0.149)
Poor upper extremity	0.536^+^	0.589^+^	0.759	0.691
	(0.135)	(0.138)	(0.129)	(0.149)
Poor lower extremity	2.995**	1.186	1.223+	1.320^+^
	(0.435)	(0.141)	(0.161)	(0.143)
Poor hearing	1.015	1.030	1.128	0.990
	(0.229)	(0.173)	(0.138)	(0.157)
	*Medium education level*			
Average prevalence	0.87%	0.98%	1.05%	1.97%
Overweight	1.485^+^	1.213**	1.258**	1.261**
	(0.256)	(0.076)	(0.060)	(0.081)
Poor general health	2.194**	1.051	1.048	1.123^+^
	(0.475)	(0.057)	(0.045)	(0.062)
Poor eyesight	1.327+	1.075	1.023	1.038
	(0.619)	(0.048)	(0.036)	(0.048)
Poor mental health	2.917**	1.326**	1.212**	1.376**
	(0.619)	(0.138)	(0.078)	(0.112)
Poor upper extremity	1.177	0.731	0.893	0.834
	(0.494)	(0.139)	(0.124)	(0.150)
Poor lower extremity	0.771	1.168	1.025	1.041
	(0.147)	(0.067)	(0.050)	(0.066)
Poor hearing	1.394	1.129	1.215	1.259^+^
	(0.398)	(0.127)	(0.137)	(0.141)
	*High education level*			
Average prevalence	0.57%	0.78%	0.64%	1.33%
Overweight	1.214	1.007	1.318+	1.436+
	(0.441)	(0.187)	(0.167)	(0.233)
Poor general health	1.926**	0.9992	0.931	1.083
	(0.442)	(0.136)	(0.109)	(0.142)
Poor eyesight	1.049	1.252^+^	1.077	1.193
	(0.189)	(0.113)	(0.077)	(0.182)
Poor mental health	9.637**	1.541^+^	1.295	1.660**
	(5.755)	(0.295)	(0.199)	(0.383)
Poor upper extremity	0.899	1.062	1.098	1.347
	(0.636)	(0.472)	(0.369)	(0.536)
Poor lower extremity	2.181^+^	1.125	1.098	1.247
	(0.792)	(0.167)	(0.132)	(0.176)
Poor hearing	1.000	0.770	1.158	1.044
	(0.578)	(0.347)	(0.281)	(0.348)

Also included are a quadratic trend in the birth date, period dummies for the home care observation year and care purchasing agency region dummies, father’s occupation and IQ-measurement. All analyses weighted by the sampling weights.

^+^*p*<0.05,**p<0.01. Household: men using household home care in 2004; Personal: men using personal home care in 2004; Nursing: men using nursing home care in 2004; Total: men using any home care in 2004.

## 5. Conclusion and discussion

This paper is the first to study the relationship between health indicators at age 18 and the use of formal home care later in life at age 57–69. In the empirical analysis, we use administrative data on a sample of Dutch men born in 1944–1947 to estimate bivariate panel probit models that account for unobserved heterogeneity and selective survival. As a further strength of the current analysis we note that home care use was evaluated taking regional variation in home care access and financing into account.

Our results indicate that poor health early in life, especially having poor mental health or being overweight at age 18, is associated with problems that require home-based formal care services later in life. Having poor general health or problems with legs or feet early in life increase the probability of health problems later in life to a much smaller degree. We show that the impact of poor lower extremities on home care use is mostly seen among men from a lower socioeconomic background (fathers with manual occupation). We also show that, for less educated men, both being overweight or being rated with poor general health at age 18 has a larger impact on home care use later in life than for medium or higher educated men.

Some specific conditions that lead to a poor health rating on physical characteristic included congenital malformations, blindness, deafness and conditions acquired during childhood (paralytic polio, bone fractures cardiac and other infections). Much progress has been made since the 1960’s on the prevention of many of these conditions.

In recent years there has been a continued policy debate about the sustainability of national long-term care provisions, due to the ageing of the population in all developed countries. In the Netherlands. Starting in 2013, several reforms have shifted an increasing part of the financial burden to households, these now have to face higher medical out of pocket expenditures for long-term care. Our results highlight the potential of interventions to diagnose and treatment of adults with mental health problems and to educate them about the long term impact of weight problems on later health.

Our study has some limitations. First, large data sets for similar analyses among women are not available in the Netherlands. Second, although the follow-up time in this study is close to 50 years, the utilization of home care at age 70 was still relatively low. Continued follow-up of the cohort will therefore be needed to monitor the utilization of home care with further aging of the study cohort. Third, we describe relations between early-life health measurements and later life home in an observational study, subject to unmeasured sources of bias. Although suggestive, the reported relations therefore fall short of providing measures of causal effects.

## Supporting information

S1 Table Average (%) health problems at age 18 by attrition status in 2004. (DOCX)

S2 Table Odds ratios non-health controls on later life home care use. (DOCX)

S3 Table Parameters panel probit model with random effects, joint model: Parameters homecare use probability. (DOCX)

S4 Table Parameters panel probit model with random effects, joint model: Parameters probit survival model. (DOCX)

## References

[1] Case A, Fertig A, Paxson C. The lasting impact of childhood health and circumstance. Journal of Health economics. 2005; 24(2):365–389. doi: 10.1016/j.jhealeco.2004.09.008 15721050

[2] Case A, Paxson C. Causes and consequences of early-life health. Demography. 2010; 47(1): S65–S85. doi: 10.1353/dem.2010.0007 21302429 PMC3730845

[3] Currie J. Healthy, wealthy, and wise: Socioeconomics status, poor health in childhood, and human capital development. Journal of Economic Literature. 2009; 41:87–122.

[4] Almond D, Currie J. Killing me softly: The fetal origins hypothesis. Journal of Economic Perspectives. 2011; 25:153–172. doi: 10.1257/jep.25.3.153 25152565 PMC4140221

[5] Almond D, Currie J, Duque V. Childhood circumstances and adult outcomes: Act II, Journal of Economic Literature. 2018; 56(4):1360–1446.

[6] Ekamper P, van Poppel F, Stein AD, Lumey LH. Independent and additive association of prenatal famine exposure and intermediary life conditions with adult mortality between age 18–63 years. Social Science & Medicine. 2014; 119:232–239. doi: 10.1016/j.socscimed.2013.10.027 24262812 PMC4242798

[7] Ekamper P, van Poppel F, Stein AD, Bijwaard GE, Lumey LH. Prenatal famine exposure and adult mortality from cancer, cardiovascular disease, and other causes through age 63 years. American Journal of Epidemiology. 2015; 181(4):271–279. doi: 10.1093/aje/kwu288 25632050 PMC4325678

[8] van den Berg GJ, Lindeboom M, Portrait FRM. Economic conditions early in life and individual mortality. American Economic Review. 2006; 96:290–302. doi: 10.1257/000282806776157740 29125729

[9] van den Berg GJ, Doblhammer G, Christensen K. Being born under adverse economic conditions leads to a higher cardiovascular mortality rate later in life: Evidence based on individuals born at different stages of the business cycle. Demography. 2011; 48:507–530. doi: 10.1007/s13524-011-0021-8 21509649

[10] Alessie RJM, Angelini V, van den Berg GJ, Mierau JO, Viluma L. Economic conditions at birth and cardiovascular disease risk in adulthood: evidence from new cohorts. Social Science & Medicine, 2019; 224:77–84. 30769195 10.1016/j.socscimed.2019.02.006

[11] Doblhammer G, van den Berg GJ, Fritze T. Economic conditions at the time of birth and cognitive abilities late in life: evidence from ten European countries PLOS ONE. 2013; 8.9:e74915. doi: 10.1371/journal.pone.0074915 24040361 PMC3770637

[12] Ben Shlomo Y, Kuh D. A life course approach to chronic disease epidemiology: Conceptual models, empirical challenges and interdisciplinary perspectives. International Journal of Epidemiology. 2002; 31:285–293. 11980781

[13] Swinkels JC, Suanet B, Deeg DJH, van Groenou MIB. Trends in the informal and formal home-care use of older adults in the Netherlands between 1992 and 2012. Ageing & Society. 2016; 36(9):1870–1890.

[14] Stein ZA, Susser MW, Saenger G, Marolla F. Famine and Human Development: The Dutch Hunger Winter of 1944–1945. Oxford UP, New York; 1975.

[15] Ravelli GP, Stein ZA, Susser MW. Obesity in young men after famine exposure in utero and early infancy. New England Journal of Medicine. 1976; 295(7):349–353. doi: 10.1056/NEJM197608122950701 934222

[16] Vasunilashorn S, Martinson ML. Weight status in adolescence is associated with later life functional limitations. Journal of Aging and Health. 2013; 25(5):758–775. doi: 10.1177/0898264313491426 23751894 PMC4552046

[17] Roodman D. Fitting fully observed recursive mixed-process models with cmp. Stata Journal. 2011; 11(2):159–206.

[18] Batty GD, David G. Early life intelligence and adult health. British Medical Journal 2004; 329(7466):585–586. doi: 10.1136/bmj.329.7466.585 15361422 PMC516648

[19] Batty GD, Deary IJ, Gottfredson LS. Premorbid (early life) IQ and later mortality risk: Systematic review, Annals of Epidemiology. 2007; 17(4):278–288. doi: 10.1016/j.annepidem.2006.07.010 17174570

[20] Deary IJ Why do intelligent people live longer? Nature. 2008; 456(7219):175–176. doi: 10.1038/456175a 19005537

[21] Cohen S, Janicki Deverts D, Chen E, Matthews KA. Childhood socioeconomic status and adult health. Annals of the New York Academy of Sciences. 2010; 1186:37–55. doi: 10.1111/j.1749-6632.2009.05334.x 20201867

[22] Zimmer Z, Hanson H, Smith K. Childhood socioeconomic status, adult socioeconomic status, and old-age health trajectories: Connecting early, middle, and late life. Demographic Research. 2016; 34:285–320.

[23] Galama TJ, van Kippersluis H. A theory of socio-economic disparities in health over the life cycle. The Economic Journal. 2018; 129(617):338–374. doi: 10.1111/ecoj.12577 30905971 PMC6430209

[24] Lindeboom M., Portrait FRM, Deeg DJH. The use of long-term care services by the Dutch elderly. Health Economics. 2000; 9:13–531. doi: 10.1002/1099-1050(200009)9:6&lt;513::aid-hec534&gt;3.0.co;2-r 10983004

[25] Conti G, Heckman JJ. Understanding the early origins of the education-health gradient: a framework that can also be applied to analyze gene-environment interactions. Perspectives on Psychological Science. 2010; 5(5): 585–605. doi: 10.1177/1745691610383502 21738556 PMC3129786

[26] Clark D, Royer H., The Effect of Education on Adult Mortality and Health: Evidence from Britain. American Economic Review. 2013; 103(6): 2087–2120. doi: 10.1257/aer.103.6.2087 29533059

[27] Bijwaard GE, van Poppel F, Ekamper P, Lumey LH. Gains in life expectancy associated with higher education in Men, PLOS ONE. 2015; 10(10), e0141200. doi: 10.1371/journal.pone.0141200 26496647 PMC4619701

