# Supplementary material for: Physical and psychological health at adolescence and home care use later in life
Source: PLoS One. 2021 Dec 8;16(12):e0261078. doi: 10.1371/journal.pone.0261078 (PMC8654204; doi:10.1371/journal.pone.0261078)
Supplement: S1 Table — (DOCX) [file pone.0261078.s001.docx]

S1 Table: Average (%) health problems at age 18 by attrition status in 2004

|  | remain | died | Other attrition |
| --- | --- | --- | --- |
| Overweight (BMI > 25) | 6*.69* | 8.55^**^ | 7.02 |
|  | (0*.*13) | (0 48) | (0*.*37) |
| Poor general health | 12.71 | 18.98^**^ | 13.52 |
|  | (0.17) | (0.69) | (0.50) |
| Poor sight | 19.48 | 21.14 | 19.46 |
|  | (0.21) | (0.71) | (0.029) |
| Poor mental health | 6.65 | 11.57^**^ | 7.49^+^ |
|  | (0.13) | (0.55) | (0.38) |
| Poor upper extremity | 2.87 | 5.74^**^ | 2.79 |
|  | (0.09) | (0.40) | (0.23) |
| Poor lower extremity | 9.87 | 12.91^**^ | 9.12 |
|  | (0.16) | (0.58) | (0.42) |
| Poor hearing | 4.09 | 8.02^**^ | 3.77 |
|  | (0.10) | (0.47) | (0.28) |

Remain: men still in the sample in 2004; died: men who died before 2004; other attrition: men who were lost before 2004 for other reasons. ^+^ p < 0.05, ^**^ p < 0.01 in comparison with remain
