# Supplementary material for: Physical and psychological health at adolescence and home care use later in life
Source: PLoS One. 2021 Dec 8;16(12):e0261078. doi: 10.1371/journal.pone.0261078 (PMC8654204; doi:10.1371/journal.pone.0261078)
Supplement: S2 Table — (DOCX) [file pone.0261078.s002.docx]

S2 Table: Odds ratios non-health controls on later life home care use, accounting for selective survival

|  | Household | Personal | Nursing | Total |
| --- | --- | --- | --- | --- |
| *Father’s occupation*  Professional | 1 | 1 | 1 | 1 |
| White collar | 0.830 | 0.996 | 1.014 | 0.967 |
|  | (0.098) | (0 044) | (0.035) | (0.043) |
| Farm owner | 0.461** | 1.005 | 0.943 | 0.847 |
|  | (0.115) | (0 083) | (0.063) | (0.074) |
| Skilled | 0.903 | 1.061 | 1.066 | 1.043 |
|  | (0.111) | (0 048) | (0.038) | (0.047) |
| Unskilled | 0.921 | 1.124+ | 1.071 | 1.087 |
|  | (0.119) | (0 059) | (0.044) | (0.057) |
| Unknown | 1.046 | 1.166+ | 1.134^+^ | 1.164^+^ |
|  | (0.161) | (0 074) | (0.057) | (0.074) |
| *IQ*  1 (highest) | 0.398^∗∗^ | 0.803^∗∗^ | 0.852^∗∗^ | 0.755^∗∗^ |
|  | (0.049) | (0.037) | (0.030) | (0.035) |
| 2 | 0.656^∗∗^ | 0.900^∗∗^ | 0.913^∗∗^ | 0.856^∗∗^ |
|  | (0.071) | (0.036) | (0.029) | (0.035) |
| 3 | 1 | 1 | 1 | 1 |
| 4 | 1.318 | 1.160^∗∗^ | 1.110^∗∗^ | 1.142^∗∗^ |
|  | (0.188) | (0.057) | (0.043) | (0.057) |
| 5 | 2.050^∗∗^ | 1.356^∗∗^ | 1.311^∗∗^ | 1.475^∗∗^ |
|  | (0.273) | (0.074) | (0.055) | (0.080) |
| 6 (lowest) | 3.133^∗∗^ | 1.620^∗∗^ | 1.226^∗∗^ | 1.676^∗∗^ |
|  | (0.422) | (0.113) | (0.072) | (0.119) |
| 9 (missing) | 1.199 | 1.212^+^ | 1.088 | 1.179 |
|  | (0.226) | (0.098) | (0.071) | (0.097) |

Also included are a quadratic trend in the birth date, period dummies for the home care observation year and care purchasing agency region dummies. Household: men using household home care in 2004; Personal: men using personal home care in 2004; Nursing: men using nursing home care in 2004; Total: men suing any home care in 2004.

^+^*p <* 0*.*05*,*^∗∗^ *p <* 0*.*01.
