# Supplementary material for: Physical and psychological health at adolescence and home care use later in life
Source: PLoS One. 2021 Dec 8;16(12):e0261078. doi: 10.1371/journal.pone.0261078 (PMC8654204; doi:10.1371/journal.pone.0261078)
Supplement: S3 Table — (DOCX) [file pone.0261078.s003.docx]

S3 Table: Parameters panel probit model with random effects, joint model:

Parameters homecare use probability

|  | Household | Personal | Nursing | Total |
| --- | --- | --- | --- | --- |
| Overweight (BMI > 25) | 0*.196* | 0.215^**^ | 0*.*240^∗∗^ | 0*.*280^∗∗^ |
|  | (0*.*111) | (0 051) | (0*.*039) | (0*.*051) |
| Poor general health | 0.476^∗∗^ | 0.037 | 0.043 | 0.113** |
|  | (0.097) | (0.044) | (0.034) | (0.043) |
| Poor sight | 0.198^+^ | 0.101^**^ | 0.025 | 0.053 |
|  | (0.087) | (0.036) | (0.029) | (0.037) |
| Poor mental health | 1.058^∗∗^ | 0.385^**^ | 0.241^∗∗^ | 0.418^∗∗^ |
|  | (0.109) | (0.055) | (0.045) | (0.056) |
| Poor upper extremity | 0.163 | -0.186 | −0.084 | −0.088 |
|  | (0.228) | (0.111) | (0.086) | (0.109) |
| Poor lower extremity | 0.477^∗∗^ | 0.120+ | 0.080+ | 0.122^+^ |
|  | (0.124) | (0.049) | (0.039) | (0.049) |
| Poor hearing | −0.377 | 0.078 | 0.176^+^ | 0.161+ |
|  | (0.223) | (0.084) | (0.064) | (0.082) |
| *Father’s occupation*  White collar | −0.187 | 0.004 | 0.014 | −0.034 |
|  | (0.118) | (0.044) | (0.034) | (0.044) |
| Farm owner | −0.775** | 0.005 | −0.059 | −0.166 |
|  | (0.250) | (0.083) | (0.066) | (0.087) |
| Skilled | −0.102 | 0.060 | 0.064 | 0.042 |
|  | (0.123) | (0.045) | (0.035) | (0.045) |
| Unskilled | 0.083 | 0.117^+^ | 0.069 | 0.083 |
|  | (0.130) | (0.052) | (0.041) | (0.052) |
| Unknown | 0.045 | 0.154^+^ | 0.126^+^ | 0.152^+^ |
|  | (0.155) | (0.063) | (0.050) | (0.064) |
| *IQ*  1 (highest) | −0.921^∗∗^ | −0.219^∗∗^ | −0.160^∗∗^ | −0.281^∗∗^ |
|  | (0.122) | (0.046) | (0.035) | (0.046) |
| 2 | −0.422^∗∗^ | −0.106^∗∗^ | −0.091^∗∗^ | −0.155^∗∗^ |
|  | (0.108) | (0.041) | (0.031) | (0.041) |
| 4 | 0.276 | 0.149^∗∗^ | 0.105^∗∗^ | 0.133^∗∗^ |
|  | (0.143) | (0.049) | (0.039) | (0.050) |
| 5 | 0.718^∗∗^ | 0.305^∗∗^ | 0.271^∗∗^ | 0.388^∗∗^ |
|  | (0.135) | (0.055) | (0.043) | (0.054) |
| 6 (lowest) | 1.142^∗∗^ | 0.482^∗∗^ | 0.204^∗∗^ | 0.516^∗∗^ |
|  | (0.135) | (0.070) | (0.059) | (0.071) |
| 9 (missing) | 0.181 | 0.193^+^ | 0.085 | 0.165+ |
|  | (0.188) | (0.081) | (0.066) | (0.082) |
| Constant | −6.426^∗∗^ | −3.856^∗∗^ | −3.100^∗∗^ | −3.620^∗^ |
|  | (0.259) | (0.091) | (0.068) | (0.086) |

Reference category: father’s occupation professional, IQ-level 3. Also included are a quadratic trend in the birth date, period dummies for the home care observation and care purchasing agency region dummies. Household: men using household home care in 2004; Personal: men using personal home care in 2004; Nursing: men using nursing home care in 2004; Total: men suing any home care in 2004. All analyses weighted by the sampling weights. ^+^*p <* 0*.*05*,*^∗∗^ *p <* 0*.*01.
