# Supplementary material for: Physical and psychological health at adolescence and home care use later in life
Source: PLoS One. 2021 Dec 8;16(12):e0261078. doi: 10.1371/journal.pone.0261078 (PMC8654204; doi:10.1371/journal.pone.0261078)
Supplement: S4 Table — (DOCX) [file pone.0261078.s004.docx]

S4 Table: Parameters panel probit model with random effects, joint model:

Parameters probit survival model

|  | | Household | Personal | Nursing | Total |
| --- | --- | --- | --- | --- | --- |
| Care in previous year | | 0*.684*^∗∗^ | 0.657^**^ | 0*.*683^∗∗^ | 0*.*705^∗∗^ |
|  | | (0*.*033) | (0.028) | (0*.*027) | (0*.*021) |
| Overweight (BMI>25) | | 0*.*098^∗∗^ | 0.102^**^ | 0*.*099^∗∗^ | 0*.*095^∗∗^ |
|  | | (0*.*023) | (0.023) | (0*.*023) | (0*.*023) |
| Poor general health | | 0*.*046^+^ | 0.056^**^ | 0*.*054^∗∗^ | 0*.*052^+^ |
|  | | (0*.*019) | (0.019) | (0*.*019) | (0*.*020) |
| Poor sight | | 0*.*037^+^ | 0.039^+^ | 0*.*041^+^ | 0*.*038^+^ |
|  | | (0*.*016) | (0.016) | (0*.*016) | (0*.*016) |
| Poor mental health | | 0*.*074^∗∗^ | 0.077^**^ | 0*.*078^∗∗^ | 0*.*062^+^ |
|  | | (0*.*026) | (0.026) | (0*.*026) | (0*.*027) |
| Poor upper extremity | | −0*.*112^+^ | -0.111^+^ | −0*.097* | −0*.*110^+^ |
|  | | (0*.*052) | (0.052) | (0*.*052) | (0*.*052) |
| Poor lower extremity | | −0*.*022 | -0.018 | −0*.*017 | −0*.*024 |
|  | | (0*.*023) | (0.023) | (0*.*023) | (0*.*023) |
| Poor hearing | | 0*.*077+ | 0.076+ | 0*.*069 | 0*.*071 |
|  | | (0*.*037) | (0.037) | (0*.*037) | (0*.*038) |
| *Father’s occupation* |  |  |  |  |  |
| White collar |  | 0*.*033 | 0.026 | 0*.*027 | 0*.*030 |
|  |  | (0*.*019) | (0.019) | (0*.*020) | (0*.*020) |
| Farm owner |  | -0*.*003 | -0.020 | -0*.*021 | -0*.*009 |
|  |  | (0*.*038) | (0.038) | (0*.*039) | (0*.*039) |
| Skilled |  | 0*.*046+ | 0.037 | 0*.038* | 0*.041+* |
|  |  | (0*.*020) | (0.020) | (0*.*020) | (0*.*020) |
| Unskilled |  | 0*.*052^+^ | 0.045 | 0*.*045 | 0*.*045 |
|  |  | (0*.*023) | (0.023) | (0*.*024) | (0*.*024) |
| Unknown |  | 0*.105*** | 0.100** | 0*.*098** | 0*.*096** |
|  |  | (0*.*028) | (0*.*028) | (0*.*029) | (0*.*029) |
|  | |  |  |  |  |

Survival till 2004 and subsequent annual survival. Reference category: father’s occupation professional, IQ-level 3. Also included are a quadratic trend in the birth date, period dummies for the home care observation and care purchasing agency region dummies. Household: men using household home care in 2004; Personal: men using personal home care in 2004; Nursing: men using nursing home care in 2004; Total: men suing any home care in 2004. All analyses weighted by the sampling weights. ^+^*p <* 0*.*05*,*^∗∗^ *p <* 0*.*01.

S4 Table: Parameters panel probit model with random effects, joint model:

Parameters probit survival model (continued)

|  | | Household | Personal | Nursing | Total |
| --- | --- | --- | --- | --- | --- |
| *IQ* |  |  |  |  |  |
| 1 (highest) |  | -0.087** | -0.091** | -0.099** | -0.088** |
|  |  | (0.020) | (0.020) | (0.020) | (0.020) |
| 2 |  | -0.050** | -0.052** | -0.057** | -0.051** |
|  |  | (0.018) | (0.018) | (0.018) | (0.018) |
| 4 |  | 0.019 | 0.018 | 0.016 | 0.015 |
|  |  | (0.022) | (0.022) | (0.022) | (0.018) |
| 5 |  | 0.076** | 0.080** | 0.077** | 0.065** |
|  |  | (0.025) | (0.025) | (0.025) | (0.025) |
| 6 (lowest) |  | 0.052 | 0.067+ | 0.085+ | 0.043 |
|  |  | (0.034) | (0.034) | (0.034) | (0.035) |
| 9 (missing) |  | 0.005 | 0.012 | 0.017 | 0.008 |
|  |  | (0.038) | (0.038) | (0.038) | (0.039) |
| *σ_ω_* |  | 2.776** | 1.214** | 0.954** | 1.530** |
|  |  | (0.065) | (0.023) | (0.018) | (0.026) |
| *Ρ^b^* |  | 0.284^∗∗^ | 0.746^∗∗^ | 0.769^∗∗^ | 0.688^∗∗^ |
|  |  | (0.026) | (0.009) | (0.008) | (0.009) |
| Constant |  | −2.310^∗∗^ | −2.308^∗∗^ | −2.325^∗∗^ | −2.341^∗∗^ |
|  | | (0.037) | (0.037) | (0.037) | (0.037) |

Survival till 2004 and subsequent annual survival. Reference category: father’s occupation professional, IQ-level 3. Also included are a quadratic trend in the birth date, period dummies for the home care observation and care purchasing agency region dummies. Household: men using household home care in 2004; Personal: men using personal home care in 2004; Nursing: men using nursing home care in 2004; Total: men suing any home care in 2004. All analyses weighted by the sampling weights. ^+^*p <* 0*.*05*,*^∗∗^ *p <* 0*.*01.
